# A Specimen of Exostosis of the Nasal Septum

**Published:** 1888-09

**Authors:** R. O. Cotter

**Affiliations:** Macon


### A SPECIMEN OF EXOSTOSIS OF THE NASAL SEPTUM.

---

*Editors Atlanta Medical and Surgical Journal :*

I inclose you herewith a specimen of an exostosis of the nasal septum which I removed from a gentleman a few days ago. This is not properly the ecchondrosis so admirably described by Carl Seiler, of Philadelphia, but true osseous substance rather than semi-cartilaginous. The size of this one, now seven days after removal and partially dry, is ⅞ inch in length, ½ inch in width and ¼ inch thick.* The specimen looks like a small thing to have been a source of trouble, but if you reflect what it was when it was not dried and denuded of its mucous tissue when in a normal condition of the latter, much less when congested and thickened by cold, dust and other irritating causes, you will readily appreciate the statement that the patient suffered with the usual symptoms of nasal occlusion, viz., catarrhal headache, snuffling and sneezing, mouth-breathing, naso-pharyngitis, etc. This had gone on for three years, and it was only lately, when he had begun to be affected with decided symptoms of middle-ear catarrh, that he became alarmed enough to come for treatment. In addition to this shell-like exostosis which I show, and which was situated on the posterior third of the left side of the nasal septum, even with a line between the inferior and middle turbinated bones, there was an ecchondrosis or partially cartilaginous hypertrophy, directly anterior to it on the same surface of the septum. This latter was, I have no doubt—for such is my usual observation—a secondary formation; that is, was a result of the posterior bony spur. These were removed with the saw. The saws I use are hardly more than a line in width, and cut with a withdrawing motion. For the specimen I inclose you, owing to its high location, I used the downward saw that there might be no danger in wounding the vault of the nostril, while for the anterior cartil-

*It was found impossible to obtain a good cut of the specimen, so an illustration cannot be given.—PUBLISHER.

aginous ecchondrosis I used the upward saw, as I usually do. The latter are, if complicated with much bony tissue, often exceedingly tedious to remove and require the aid of knives, scissors, etc. I find the double-edged curved knife devised by Carl Seiler to be a very useful adjuvant. I have done some thirty odd of these sawing operations, and last December I had Dr. F. H Bosworth, of New York, remove with the saw a large hypertrophy from my own nasal septum, and so am prepared to speak personally as well as professionally of what I know. I have nothing but the highest praise for and the utmost satisfaction in the results of what I have seen of these operations. All I have seen have healed kindly and very quickly. None have shown any tendency to return. They have totally or to a very great extent removed the unpleasant symptoms for which the operations were employed. I have certainly seen very many cases of aural catarrh and naso-pharyngitis result from neglect of removal of these hypertrophies. Of course I will explain that I did not mean to claim that *old cases of middle-ear catarrh*, the result of these causes, would yield to treatment upon the removal of the latter. There is one kind of nasal septum trouble which I have not thought it advisable to operate upon with the saw, or in fact to attempt to remove at all. These are simple *deflections without hypertrophy* of the septum. My experience is that these are generally the result of blows upon the nose. Although they will and do often almost entirely occlude the nostril, my reason for not operating on them is on account of the possibility of too much destruction of osseous tissue already sparsely supplied with blood, which might lead to caries of the septum; yet, Dr. Seiler, of Philadelphia, writes me that he doesn't hesitate to operate upon these. I use a spray of a 10 % solution of cocaine. I gave my reasons fully for not using a 20 % in an article on cocaine in the July (1888) number of THE ATLANTA MEDICAL AND SURGICAL JOURNAL. I may add, in conclusion, that the pain from this operation is not to be considered as an objection to its performance.

Very truly,

R. O. COTTER, M. D.

Macon, July 20, 1888.

